# Spasm of Larynx1Read at the meeting of the Illinois State Veterinary Medical Association, Feb. 15, 1899.

**Published:** 1899-05

**Authors:** C. E. Hollingsworth

**Affiliations:** La Salle, Ill.


### REPORTS OF CASES.

### SPASM OF LARYNX.[1]

By C. E. Hollingsworth, V.S.,
LA SALLE, ILL.

The second case which I will bring before you was a six-year-old grade draught-mare, of 1400 pounds weight, in good condition, which was brought to me on January 12, 1899, with head hanging low, distended nostrils, a very anxious expression, loud and difficult respiration (almost whistling), and perspiring freely.

It was evident at a glance that the seat of the trouble was in the anterior portion of the respiratory tract, and not in the lungs, as the owner insisted; but to satisfy him I examined them, with negative results.

Asking for the history of the case, he stated that the first attack was in November, as they were gathering corn; the mare became frightened and tried to run away, when she was seized with the symptoms here presented, but with a half-hour's rest she would be apparently all right again; but a week or two later had a return attack, resulting from the excitement caused from fright. When not used, and especially when not excited, there was no indication of anything wrong.

I suspected nasal polypus, but after an examination, made as carefully as possible, considering the extreme restlessness of the patient, I could find nothing. Then, again listening to the whistling sounds, the greatest noise came from the region of the larynx, thus furnishing, to my mind, conclusive evidence that it was spasm of the larynx, due to nervous excitability.

[1] Read at the meeting of the Illinois State Veterinary Medical Association, Feb. 15, 1899.

As she was suffering great inconvenience, I recommended tracheotomy as the best and quickest means of relief. But the owner refused, saying she would be all right in twenty minutes; in fact, that she was better already, and that if there was no trouble in her lungs there must be a piece of cornstalk lodged in her nostril. I explained the utter impossibility of that, without there being some local symptoms as result, and added that it would take a microscope to see that she was any better, and that she was in danger of dying from asphyxia.

The dyspnœa was now very much increased, a frothy saliva exuding from the mouth, pulse quick and weak, staring eyeballs—all the symptoms being much more pronounced. After his refusal to permit me to operate I tried chloroform inhalations, which, however, only aggravated the symptoms, though it was very carefully administered.

Seeing that she would soon fall, I prepared for emergencies, and none too soon, as she began to reel and stagger about the stall, and fell before anything could then be done to prevent. She arose, and fell again very quickly, this time with her head in a corner. I then assumed the offensive, and ordered them to drag her around until I could get at the trachea, when, with a slash, I bared the cartilaginous rings for about six inches, haste being of more import just then than science. I then severed two or three of the rings, holding the divided edges apart with my fingers, so that respiration could be accomplished while they were bringing me the tube—this being an absolute necessity—as she was so far gone that respiration had ceased, likewise all struggles, and the eyes had assumed that characteristic appearance with which some of us are only too familiar.

As soon as the rings were severed she began to breathe, and within one minute was on her feet, respiration being performed with ease through the tube.

The next day she was taken five miles toward home, remained there four or five days, and then completed the journey home, which was twelve miles in the country.

I regret to say that I have heard nothing further from the case. My instructions were to use the tube until, under all circumstances which had previously caused these attacks, she experienced no difficulty, then bring her back and I would close the wound.

He did not wish any medical treatment. My opinion is that nature, unassisted, will eventually overcome the derangement; but I think proper medication would materially hasten recovery.

